# The genus *Cuscuta* (Convolvolaceac): An updated review on indigenous uses, phytochemistry, and pharmacology

**DOI:** 10.22038/ijbms.2019.35296.8407

**Published:** 2019-11

**Authors:** Shazia Noureen, Sobia Noreen, Shazia Akram Ghumman, Fozia Batool, Syed Nasir Abbas Bukhari

**Affiliations:** 1 Department of Chemistry, University of Sargodha, Sargodha-40100, Pakistan; 2 College of Pharmacy, University of Sargodha, Sargodha-40100, Pakistan; 3 Department of Pharmaceutical Chemistry, College of Pharmacy, Jouf University, Aljouf, Sakaka2014, Saudi Arabia

**Keywords:** Bioactive, Cuscuta, Folk medicines, Pharmacological activities, Phytochemicals

## Abstract

*Cuscuta*, commonly known as dodder, is a genus of family convolvolaceace. Approximately 170 species of *Cuscuta* are extensively distributed in temperate and subtropical areas of the world. Species of this genus are widely used as essential constituents in functional foods and traditional medicinal systems. Various parts of many members of Cuscuta have been found efficacious against a variety of diseases. Phytochemical investigations have confirmed presence of biologically active moieties such as flavonoids, alkaloids, lignans, saponines, phenolics, tannins, and fatty acids. Pharmacological studies and traditional uses of these plants have proved that they are effective antibacterial, antioxidant, antiostioporotic, hepatoprotective, anti-inflammatory, antitumor, antipyretic, antihypertensive, analgesic, anti hair fall, and antisteriogenic agents.

## Introduction

Plant-based medicines are an integral part of virtually all cultures since immemorial times. The journey of information from prehistoric texts to various indigenous folklores and modern preparations have witnessed the presence of bioactive moieties with therapeutic potential in these herbs (1-4). The immense population of current allopathic products is embedded in nature. More than half of the clinically approved drugs in the world are either natural products or their modifications. Higher plants being an endless reservoir contribute above one fourth. The remarkable resurgence of interest in nature to explore pharmaceutical and nutraceutical agents is still marching towards new horizons (5-7). 

Ever growing consumption of natural products by local masses has forcefully motivated the scientists to acquire systematic, elaborated, and practical knowledge about their constituents by using advanced technologies (8). Herbal products, both as purified compounds and in the form of standard extracts, offer infinite odds for novel pharmaceutical products due to the matchless accessibility to different chemical species (9). Target-based phytochemicals have transfigured the medicinal industry because these are not only directly utilized for treatment purposes but also act as leads and standard template for synthetics drugs (10-11). Therefore, modern scientific investigations are turning towards traditional medicines to look for new windows of opportunities giving rise to superior pharmacologically active agents against diseases (12). 

The genus *Cuscuta *L. commonly known as dodder is one of the essential herbal constituents of pharma foods and curative tonics that are frequently prescribed to nourish various body parts. It is used to enhance the nutritional value of porridge and alcoholic beverages (13). The genus has a rich history of folk medicinal uses, and numerous phytoconstituents of therapeutic value have been isolated and identified (14). Various species are indigenously used to cure fits, melancholy, insanity (15), fertility problems (16), tumors (17), scabies, eczema (18), chronic ulcer, jaundice, inflammation (19), chest pain (20), fever, itching (21), osteoporosis (22), diarrhea, oedema, stomach ache, infections, measles, sores, kidney problems (23), sprain (24), alleviation of high blood pressure, leucorrhoea (25), obesity (26), migraine, amnesia, epilepsy, and constipation (27).

Pharmacological analysis of various *Cuscuta* species unveiled their antitumor, antimicrobial (28-31), hepatoprotective (32-33), anticonvulsant (34), immunostimulatory, antioxidant (14, 35-37), α-glucosidase inhibition (38), psychopharmacological (39), hair-growth promoting (40-41), anti-steroidogenic (42), anti-inflammatory (43-44), diuretic (45), analgesic (46), antipyretic (47-48), anti-HIV (49), antidiabetic (50), neuroprotective (51), antiulcer (52), antispasmodic, heamodynamic, bradycardia1, antihypertensive, cardiotonic, and muscle relaxant activities (53).

*Cuscuta *species are rich in bioactive constituents that exhibit a wide variety of pharmacological activities. Presence of a good deal of valuable components, broad range of biological attributes and remedial value of these plants in folk medicinal systems gives stimulation toward the concept that this genus can play an important role in discovery of new and more efficient therapeutic agents. This review is an effort to edify knowledge of its phytochemical richness, pharmacological and biological significance, and folk medicinal uses, which will enhance its value as a potent pharmaceutical precursor. 

## Methods

This review on *Cuscuta* genus has been written according to the information collected from various scientific databases such as Scopus, Researchgate, Web of Science, ScienceDirect, and PubMed up to August 2018.

**Distribution and botanical description**

*Cuscuta*, a flowering parasitic genus was previously placed in the *Convolvulaceae* family, but later it was segregated as the separate family *Cuscutaceae *(54-57). Global distribution record indicates that most of the species are concentrated in tropical and subtropical areas and fewer in temperate regions. This parasitic genus is known by many common names such as dodder, gold-thread, hair-weed, devil’s hair, hell-vine, strangle-vine, love-vine, pull-down, etc. in different regions of the world. The number of species documented by various authors varies from 100 to 170 (58-66). Medicinally important species are *C. reflexa *Roxb. (67),* C. chinesis *Lam. (68)*, C. japonica *Choisy (69)*, C. australis *R. Br. (70),* C. europaea *Linn. (71), *C. gigantea *Griff. (72),* C. hyalina *Roth. (73),* C. campestris *Yuncker. (47)*, C. racemosa *Mart. (52)*, C. pedicellata *Ledeb. (74),* C. epithymum *L. (75),* C. kilimanjari *Oliv. (76), *C. kotschyana *Boiss. (77), *C. mitraeformis* Engelm. (78), *C. tinctoria *Mart (79), and *C. capitata *Roxb (80).

*Cuscuta* species are holophrastic, annual or perennial, herbaceous vines. The thread-like slender, twining stems have orange, red, or yellow color. Majority of the members have achlorophyllous, scaly leaves while some of them are with reduced synthetic apparatus and can perform localized and limited photosynthesis. Bisexual flowers in multiple colors like cream, yellow, white, and pink are pollinated by insects. Roots are absent, and haustoria are used to suck water and nutrients. Several morphological and physiological simplifications, for instance absence of cotyledons or radicles in their embryos, scaly leaves without vascular tissue and haustoria represent an adaptation to parasitism. They are obligate parasitic plants (54, 61, 81-84). These stem and leaf parasites depend entirely on their host plant, thus reducing the growth and yield of the host. They mostly infect many broadleaf crops, ornamentals plants, weeds, and a few monocot crops. Some of the species are strictly host-specific while others thrive on diverse hosts (85, 86). The usual growing season is early summer; germination starts in May, parasites invade the host by haustoria and may wither and die in the absence of a suitable host within two weeks (87). Flowering starts in June and seed production in November (88). 

**Table 1:** Common names and global distribution of some medicinally important Cuscuta species

**Name **	**Common name**	**Distribution **	**References **
*C. reflexa*	Hell weed, devil's gut, beggar weed, strangle tare, scald weed, dodder of thyme, greater dodder, lesser dodder	Pakistan, India, China, E. Asia, Afghanistan, Bangladesh,	(27, 29, 89-90)
*C. chinesis*	Chinese dodder	Ethiopia, Kazakhstan, Kyrgyzstan, Tajikistan, Turkmenistan, Uzbekistan, Mongolia; Russia, China, Iran, Iraq, Afghanistan, India, Sri Lanka, Indonesia, Korea, Japan, Taiwan, Thailand, Australasia,	(68, 91)
*C. japonica*	Japanese dodder	Korea	(92-93)
*C. australis*	Australian dodder, Omonigelegele, southern dodder	Taiwan, Africa, Japan, Australia, Madagascar, Europe, Asia, Senegal, Ethopia,	(23, 70, 94-96)
*C. europaea*	**…………….**	India, Romania, Bulgaria, Iran	(97-99)
*C.* *gigantea*	**……………..**	Pakistan, China, Afghanistan, Tajikistan.	(62, 72)
*C. * *hyaline*	**……………..**	Pakistan, Ethiopia, Sudan, Kenya, Uganda, Burundi, Rwanda, Zimbabwe,India,Botswana, Namibia, South Africa,	(100)
*C. * *planif'lora*	Small seed dodder, red dodder	North Africa, South western and southern Asia, Ethiopia, Madagascar, Angola	(23, 101-102)
*C. campestris*	Field dodder, common dodder, prairie dodder, yellow dodder, gewone dodder,	Saudi Arabia, Nigeria, South America, Europe, Asia, Africa, Australia, Taiwan	(81, 86, 103-105)
*C. racemosa*	Chilean dodder, lead-vine, golden thread	Brazil, Chile	(52, 106)
*C. pedicellata*	Clover dodder	Pakistan, Egypt, Qatar, Saudi Arabia, UAE, Iran	(26, 99, 107-109)
*C. * *epithymum*	Common dodder, Clover dodder, lesser dodder, flax dodder	Pakistan, Ireland, Iran, Poland	(95, 106, 110-112)
*C. kilimanjari*	Dodder	Sudan, Etopia, Congo, Malawi, Zimbabwe, Mozambique, Limpopo, Madagascar	(23,95)
*C. monogyna*	Eastern dodder	Iran	(113)
*C. approximata*	Alfalfa dodder Smooth seed alfalfa dodder	Turkey, Iran	(14, 114-115)
*C. kotschyana*	**……………..**	Iran,	(99)
*C. capitata*	**……………..**	India, Nepal	(80,116)
*C. mitraeformis*	**.…………….**	México	(78)

**Table 2:** Traditional medicinal uses of some Cuscuta species

**Species **	**Plant part**	**Preparation **	**Traditional use**	**References **
*C. reflexa*	WP*	Paste	Treatment of swollen testicles, gout and joint pain, causes abortion, anti rheumatic, analgesic	(67, 125, 127-128, 132, 169-170)
		Maceration	Infection treatment	(149)
		Infusion	Anti-poisonous	(142)
		Juice	Antiseptic, useful in itching skin and jaundice	(127, 171)
		Powder	Anti-fertility agent, astringent, diaphoretic	(136)
		Pills	Anti tuberculosis	(89)
		Decoction	Useful in skin disease, used for jaundice, cough, blood purification, bronchitis, fever, sex stimulation	(171-172)
		---------	Anti diarrheal, anti inflammatory, anti ulcer, purgative, antidandruff, cojunctivitis, analgesic , hepatoprotective, useful in cough, cephalagia, fever, leucorrhoea, and paralysis, respiratory disorders, piles, stomach problem, constipation, spleen diseases, helminthiasis, fracture joining	(124, 131, 144, 150, 169, 173-175)
	Stem	Decoction	Hepatoprotective, anti diarrheal, useful in constipation, stomach disorders, urinary tract infections, jaundice, epilepsy, cholera, asthma	(144, 169)
		Paste	Anti-hair fall, anti rheumatic, useful in skin diseases	(29, 128, 144)
		Juice	Jaundice treatment	(126, 176)
		Crushed	Blood purifier, purgative, good for brain, fever, anthrax in cattle	(135, 138)
		---------	Effective in bilious disorders and fever	(133-134)
	Seeds	Decoction	Cause abortion	(144)
		---------	Carminative, anthelmintic, alterative, emmenagogue, sedative, diuretic, useful in ulcer, liver disorders	(129, 170)
		Poultice	Pain reliever	(177)
	Leaves	Extract	Cold treatment	(178)
		Juice	Anti hypertensive, anti diarrheal, useful in jaundice.	(179)
		---------	Effective in scabies, eczema, inducing sterility	(18, 180)
	Fruits	---------	Antipyretic, cough reliever	(67)
*C. chinensis*	WP	Juice	Anti ulcer, anti inflammatory, wound healer, jaundice treatment	(19)
		Dressing	Useful in painful inflammations	(151)
		Paste	Anti ulcer and wound healer	(151)
	Seeds	---------	Carminative, tonic, diuretic, sedative, diaphoretic	(158)
	Stem	Paste	Joining fractures	(155)
		---------	Expectorant, carminative, tonic, anthelmintic, purgative, diaphoretic, anti inflammatory, analgesic	(158)
*C. japonica*	Leaves	---------	Antihypertensive	(93)
*C. australis*	---------	---------	Laxative, anthelmintic, astringent, emollient, sedative, sudorific, liver and kidney tonic, useful in sores and measles	(23)
*C. austrais *	seeds	Decoction	Brain tonic	(181)
*C. europaea*	Sap	---------	Carminative	(71)
	WP	Extract	Anti psoriasis	(71)
		Juice	Useful in skin diseases	(167)
	Seed, vegetative pant	---------	Laxative, diuretic, analgesic	(116, 166)
*C.* * gigantea*	---------	Juice	Antipoisnous	(72, 164)
	---------	---------	Anti-septic	(116)
*C. * *hyalina*	WP	---------	Purgative, useful externally against itching and internally in protracted fevers	(21)
		Infusion	sores washers	(21)
		---------	Abortion treatment	(73)
		---------	Antiulcer, against culex mosquito,	(23)
C. *planif'lora*	WP	---------	Carminative, laxative	(130)
	Stem	---------	Anti diarrheal	(23)
*C. campestris*	WP	Decoction poultice	Purgative, useful in constipation,	(105)
*C. racemosa*	---------	---------	Anti-inflammatory, diuretic, effective in stomach and hepatic disorders and fresh wounds	(52)
*C. pedicellata*	---------	---------	Anti-obesity	(26)
	Stem	---------	Purgative, wound healer, anti-inflammatory, antihypertensive, useful in Stomachache	(168)
C. *epithymum*	WP	---------	Diuretic, laxative, liver and kidney tonic, to treat sciatica, scurvy and scrofula derma	(163, 182)
		---------	Astringent, Laxative, detersive	(75)
		Extract	Scleroderma treatment	(162)
	Stem	---------	Useful in epilepsy	(183)
*C. kilimanjari*	Stem	Sap	Useful inear, nose and throat diseases	(76)
	WP	---------	Effective in stomach ache, oedema, veterinary treatment, agalactia	(23)
	---------	Sap	Treatment of ring worm and warts	(79)
*C. capitata*	WP	Powder	Reduces irritation of bladder and improves urinary function	(80)
			Useful in kidney problem	(116)
*C. approximata*	WP	---------	Laxative, carminative, hepatoprotective	(130)
	---------	---------	Useful in sin disease	(116)

C: Cuscuta; *Whole plant

**Table 3:** Phytochemical profile of various Cuscuta species

**Name **	**Plant part **	**Solvent **	**Extraction **	**Separation** **technique **	**Phytochemicals **	**Refere-nces **
*C. reflexa *	WP	MeOH	Maceration	CC	7’-(3’,4’-dihydroxyphenyl)-N-[(4-methoxyphenyl)ethyl]propenamide 7’-(4’-hydroxy,3’-methoxyphenyl)-N-[(4-butylphenyl)ethyl]propenamide 6,7-dimethoxy-2H-1-benzopyran-2-one 2-(3-hydroxy-4-methoxyphenyl)-3,5-dihydroxy-7-O-β-D- glucopyranoside-4H-1-benzopyrane-4-one, 3-(3,4-dihydroxyphenyl)-2-propen-1-ethanoate 6,7,8-trimethoxy-2H-1-benzopyran-2-one 3-(4-O- β-D-glucopyranoside-3, dimethoxyphenyl) -2-propen-1-ol	(38)
		--------	--------	HPLC	Kaempferol Quercetin Lupeol ß-sitosterol	(215)
		Aq. EtOH	Soxhlet	TLC	Gallic acid Quarcetin	(53)
		EtOH	--------	VLC	Odoroside H 21-hydroxyodoroside H Neritaloside Strospeside 16-_-hydroxydigitoxin N-trans and cis feruloyl tyramines Ethyl caffeate Coumarins Ursolic acid _-sitosterol Glucoside 4-O-p-coumaroyl-_-D-glucoside	(216)
		n-hex	Soxhlet	GC-MS	Heneicosanoic acid Pentadecanoic acid Hexadecenoic acid Heptadecanoic acid Octadecanoic acid	(217)
	Stem	EA	Maceration	GC-MS	1, 2, 3 Propanetriol, 1- acetate, Benzofuran 2, 3, dihydro Glycerol 1, 2- diacetate 1 H- 1, 2, 4-triazol-5-amine 1- ethyl- 2-methoxy-4-vinylphenol Triacetin D - glucitol, 4 - O-hexyl 3,4,5-trimethoxy cinnamic acid Hexadecanoic acid, ethyl ester 3,6 -di methoxy phenanthrene 3, 5 - di - tert -Butyl -4 -hydroxyanisol Vanillin 3 – aminopyrrolidine Cetene Sarcosine, N -isobutyryl, tetradecyl ester 4 - ((1E) – 3 – hydroxyl -1-propenyl)-2-methoxy phenol 1,5-diphenyl-2H-1,2,4-triazoline-3-thione 1-octadecene Heptanamide, N-(1-cyclohexylethyl)-2-methyl Scoparone Hexadecanoic acid, ethyl ester 3’-Methyl-2-benzylidene-coumaran-3-one	(218)
		Pet Eth	Soxhlet	CC	5-hydroxy-7-methoxy-6-(2,3-epoxy-3-methylbutyl)-flavanone (reflexin)	(206)
					Isorhamnetin Isorhamnetin-3-O-glucoside Isorhamnetin-3-O-robinobioside	(122)
		MeOH	Maceration	GC-MS	2-Methoxy-4-vinyl phenol Benzofuran-2,3-dihydro 3,5-di-tert-Butyl-4-hydroxyanisole Hexatriacontane n-Hexadecanoic acid Scoparone Hexadecanoic acid methyl ester 1,3-Benzenediamine, N, N, N′, N′ tetramethyl- Phenol, 4(3-hydroxy1propenyl), 2-methoxy Phenol, 2,4 bis (1,1dimethylethyl); 2,3,5,6-Tetramethyl para phenylene diamine Retinoic acid-5,6-epoxy-5,6-dihydro 2,4-Dihydroxy- 2,5-dimethyl-3(2H) furan-3-one 2,3-dihydro-3,5-dihydroxy-6-methyl-2-Propyl-tetrahydro-pyran-3-ol Pregn-4-ene-18-oic acid	(219)
	AP	MeOH	Maceration	Reverse phase HPLC	Swarnalin Cis-swarnelin Coumarin 5, 6, 7-trimethoxycoumarin	(205)
	------	Water	--------	---------	Aromadendrin Taxifolin Aromadendrin-7-0-β-D-glucopyranoside 3,5,7,8,4'-pentahydroxyflavanone Taxifolin-7-0-β-D-glucopyranoside Coccinoside B Pruning 3-O-dicaffeoyl quinic acid 3-4-O-dicaffeoyl quinic acid 3, 4, 5-O-Tricaffeoylquinic acid	(49)
	-------	DCM	Maceration	HPLC	Violaxanthin Lutein Lycopene β, ψ-carotene Rubixanthin β, β – carotene Esterified rubixanthin Lutein violaxanthin β-cryptoxanthin	(220)
	Fil.	Water	Maceration	CC	An antiviral protein with molecular weight about 14,000---18,000 daltons	(219)
*C. chinesis*	Fruit	50 % MeOH	-----------	CC	Cuscutamine Cuscutoside A (2′-hydroxyl asarinin 2′-O-β-D-apiofuranosyl-(1 → 2)-β-D-glucopyranoside Cuscutoside B (2′-hydroxyl asarinin 2′-O-β-xylopyranosyl-(1 → 6)-β-glucopyranoside Hyperoside Astragalin Quercetin Quercetin-3-O-apiosyl (1-2)-galactoside Pinoresinol-4-O-glucoside Einoresinol Epipinoresinol) p-coumaric acid Caffeic acid Chlorogenic acid Arbutin	(194)
	Stem	Pet. eth CF	--------	---------	β-sitosterol d-sesamin 9(R) - hydroxy-d-sesamin D-pinoresinol daucosterol	(221)
	Seed	Pet. eth	Reflux	CC	Cuscutoside C (2′-hydroxyl asarinin 2′-O-β-D-glucopyranoside) Cuscutoside D (2′-hydroxyl asarinin 2′-O-β-D-apiofuranosyl-(1 → 2)-[β-D-glucopyranosyl-(1 → 6)]-β-D-glucopyranoside	(196)
		---------	--------	---------	Neo-sesamin Kaempferol Kaempferol-3-O-β-D-glucopyranoside 4', 4, 6-trihydroxyaurone Quercetin Hyperoside Palmitic acid Stearic acid β-sitosterol Daucosterol	(210)
		---------	--------	Reverse phase liquid chromatography	quercetin 3-O-β-D-galactoside-7-O-β-D-glucoside quercetin 3-O- β-D-apiofuranosyl-(1etin3D-galactoside hyperoside quercetin kaempferol	(218)
		Ether Water	Saponi-fication	CC	A trisaccharide Four new glycosidic acids (cuscutic acids A-D ) Acetic acid Propionic acid 2-methylbutyric acid Tiglic acid Nilic acid Convolvulinolic acid Jalapinolic acid	(194)
		95 % EtOH	--------	CC	Cuscutaresinols A−C (+)-sesamin (+)-xanthoxylol 9-hydroxysesamin (+)-pinoresinol Kaempferol Isorhamnetin	(212)
		95 % EtOH	--------	CC	Kaempferol Quercetin astragalin, isorhamnetin hyperoside	(22)
		n-hex	--------	Capillary GC	Sixteen fatty acids including Palmitic acid Linoleic acid Oleic acid Linolenic acid	(222)
		MeOH	--------	---------	Methyl 4-hydroxy-3,5dimethoxycinnamate, Caffeic acid Quercetin Kaempferol Calycopteretin	(223)
		EtOH	--------	CC	neocuscutosides A, B and C	(224)
	------	---------	--------	---------	Octadecyl (E)-p-coumarate Methyl 3-O-β-D-glucopyranosyl-5-hydroxycinnamate Quercetin-3-O-(6″-galloyl) β-D-glucoside Kaempferol Astragalin Hyperoside Astragalin 6″-O-gallate β-sitosterol Daucosterol	(225)
*C. japonica*	Seed	MeOH	--------	FCC	3, 5-Di-O-caffeoylquinic acid 3, 4-Di-O-caffeoylquinic acid Methyl 3, 5-Di-O-caffeoylquinate Methyl 3, 4-Di-O-caffeoylquinate	(226)
*C. australis*	Stem	80 % acetone	--------	CC	α-caroten-5 6-epoxide β-and γ-carotene Xanthophylls Taraxanthin Lutein Kaempferol	(227)
	Seed	---------	--------	GC	Cuscutic acids A_1_−A_3_ Acetic acid Isobutyric acid 2-methylbutyric acid Tiglic acid Nilic (3-hydroxy-2-methylbutyric) acid	(217)
		---------	--------	---------	β –sitosterol Sesamin Hexadecanoic acid Hexadecanoic acid Kaempferol Quercetin Astragloside Hyperoside caffeic acid Quercetin-3-O-β-D-galactopyranosyl-β-D-apiopyranoside	(228-229)
*C. europaea*	------	---------	--------	---------	Glycoside Flavonoids	(166)
*C. campestris*	AP	MeOH	Maceration	HPLC	Sinapic acid Quercetin Hesperidin Eugenol	(14)
*C. racemosa*	WP	70 % EtOH	Percolation	TLC	Flavonoids Tannins Flavonol (4’methoxyquercetin)	(214)
		MeOH	Socked	DCCC	Kaempherol Quercetin Pinoresinol 9-α-hydroxysesamin 9-β-hydroxysesamin Acuminatolide	(230)
		---------	--------	---------	Quercetin 5, 7, 3’, 4’-tetramethyl ether	(231)
*C. pedicellata*	WP	EtOH	--------	CC	Naringenin Kaempferol Aromadenderin Quercitin 3,5,7,30,50-pentahydroxy flavanone, Naringenin -7-O-b-D-glucoside Aromadenderin -7-O-b-D-glucoside, Taxifolin -7-O-b-D-glucoside, Kaempferol -3-O-b- D-glucoside Quercitin -3-O-b-D-glucoside	(26)
	Seed	Pet. eth	Soxhlet	CC	Quercetin Kaempferol Genkwanin Astragalin Palmitic acid	(232)
C. *epithymum*	WP	MeOH	Soxhlet	---------	Alkaloids Carbohydrates Flavonoids Glycosides Phytosterols Triterpenoids	(182)
*C. approximata*	AP	MeOH	Maceration	HPLC	Gallic acid Catechin Caffeic acid Chloregenic acid, Quercetin Coumarin,Vanilin, Eugenol	(14)
*C. monogyna*	AP	MeOH	Maceration	HPLC	Sinapic acid Catechin Caffeic acid Chloregenic acid Rutin Coumarin Vanilin Hesperidin Ellagic acid	(14)
*C. mitraeformis*	Stem	n-hex	--------	GC-FID GC-MS HPLC-DAD	Nonanal Thymol Eugenol β- carotene Lutein	**(** 78)
*C. kotschyana*	------	--------	--------	--------	Quercetin kaempferol	(233)

C: *Cuscuta; *WP: whole plant; AP: aerial parts; Fil: filament; Aq: aqueous; MeOH: methanol; EtOH: ethanol; Pet. eth: petroleum ether; n-hex: n-hexan; EA: ethyl acetate; DMC: dichloromethane; CC: column chromatography; HPLC: high performance liquid chromatography; RHPLC: reverse phase high performance liquid chromatography; TLC: thin layer chromatography; VLC: vacuum liquid chromatography; GC-MS: gas chromatography-mass spectrometry; FCC: Flash Column Chromatography; DCCC: Droplet counter-current chromatography; FID: flame ionization detector; DAD: diode array detector

**Table 4:** Pharmacological attributes exhibited by *Cuscuta* species

**Species **	**Activity **	**Plant part**	**Method **	**Extract type**	**Test applied**	**Testing model **	**Effective dose/conc.**	**Refe-rence**
*C. reflexa*	Antioxidant	St	Soxhlet	MeOH	DPPH and FRAP assay	-------------	600 μg/ml	(35)
		L		EtOH	Non-Enzymatic Glycolysation of Haemoglobin	Haemoglobin	------------	(238)
		Fl	----------	MeOH	DPPH assay	-------------	------------	(323)
	Antibacterial	L	Soxhlet	50 % EtOH	Disc diffusion method	*E. coli* *S. aureus*	------------	(29)
		St	----------	MeOH	Cup plate method	*S. aureus* *E. coli* *B. punilus* *S. typhi * *S. thyphimurium * *S. boydii * *S. sonnei * *S. dysenteriae * *P. aeruginosa* *K. pneumoniae * *V. cholerae*	125 μg/ml	(259)
		WP	----------	DCM pet. eth	Disc diffusion method	*B. subtilis * *S. lutea * *X. campestris * *E. coli* *K. pneumoniae P. vulgaris * *P.* *denitrificans*	16 to 512 μg/mL	(235)
			Soaked	EtOH	Agar well diffusion assay	*B. subtilis * *S. aureus* *E.coli * *S.typhi*	500 μg/mL	(324)
		-------	----------	MeOH	Agar well diffusion	*S. epidermidis, * *S. aureus* *E.coli Pseudomonas sp. * *K. pneumoniae*	-----------	(260)
	Antifungal	L	soxhlet	50 % EtOH	------------------	*A. niger* *C. albicans*	-----------	(29)
		-------	----------	Water	well diffusion method	*A. alternate * *A. niger* *F. solani* *F.oxysporium * *M. phaseolina*	30 % (w/v)	(107)
	Antihyperten-sive	WP	Socked	EtOH	-----------------	Wistar rats	0.1ml bolus injection	(283)
	Psycopharm-acological effect	St	Soxhlet	Pet. eth	General and exploratory behavior study	Swiss albino mice	-----------	(39)
	Anti-inflammatory	St	Suc. Ex	MeOH Pet. eth	Membrane stabilizing activity	Red blood cells	-----------	(44)
			Soxhlet	EtOH water	Percentage volume reduction	Albino rats	200, 400 mg/kg	(254)
		WP	Decoc.	Water	SQ-RT-PCR analysis	Murine macrophage cell line RAW264.7	--------------	(253)
	Diuretic Activity	AP	----------	EtOH water	Urine volume and electrolyte content	Wister rats	300 mg/kg	(45)
	Hepatoprot-ective	WP	Suc. Ex	Aq.	Biochemical parameters	Albino rats	200 mg/kg	(242)
		AP	Soxhlet	Methanol	biochemical parameters	Albino **rats**	--------------	(32)
	Antitumor /anticancer	WP	Suc. Ex	MeOH CF	------------------	Swiss albino mice MCF-7 cancer cell line	40 mg/kg	(15)
			Decoc.	Water	MTT assay DAPI staining, annexin V staining SQ-RT-PCR analysis	Human hepatocellular carcinoma cell line Hep3B	-------------	(253)
		-------	----------	CF	Annexin V-FITC apoptotic assay, PARP cleavage, Caspase activation	Hep 3B cell line	-------------	(325)
	Antisteroid-ogenic	St	Soxhlet	MeOH	Ovary and uterus weight Biochemical parameters	Swiss albino mice	-------------	(42)
	Hair growth	St	Soxhlet	Pet. eth	Visual observation Skin biopsy	----------------------	2 % extract in vehicle	(40)
					Histopathological observation	Swiss albino rats	250 mg/kg	(313)
	Antidiabetic	St	Macera.	MeOH CF	oral glucose tolerance test	Long Evans rats and Swiss albino mice	50-200 mg/kg bw	(50)
		AP	Macera.	MeOH water	oral glucose tolerance test	Swiss albino rats	400 mg/kg	(245)
	Antimutagenic	St	Soxhlet	MeOH	Ames test	*Salmonella typhimurium *TA 98 and TA 100	-------------	(122)
	Anthelmintic	WP	----------	Pet. eth CF MeOH	------------------	Pheritima posthuma	20-50 mg/ml	(44)
	Anxiolytic effect	WP	Macera.	MeOH	Elevated plus maze Light and Dark chamber	*Swiss albino mice*	400 mg /kg	(305)
	Anti-arthritic	St	----------	70 % MeOH	Percentage inhibition of oedema percentage inhibition of protein denaturation	Sprague–Dawley rats	600 mg/kg	(321)
	Nephroprote-ctive	St	----------	70 % MeOH	Biochemical parameters and pathological syptoms	Sprague–Dawley rats	600 mg/kg	(321)
	Anticonvulsant	L	Macera.	EOH	Delay the onset of convulsions	Albino mice	200 and 400 mg/kg	(238)
	Genotoxic effects	-------	----------	MeOH	root growth, root apical meristem mitotic index (MI), chromosomal aberrations	*Allium cepa *L. *Allium sativum* L.	-------------	(326)
	anti-histaminic	------	---------	EtOH		Albino rats	100 mg/kg	(327)
*C. chinensis*	Anticancer	WP	Soaked	Water	Histological study	Swiss albino mice	1 g/kg	(30
	Neuronal differentiation	Sd	Percola.	MeOH	Neurite assay	Rat pheochromocytoma PC12 cells	200 mg/L	(277)
	Adjuvant effect	Sd	----------	70 % EtOH	Splenocyte proliferation assay Indirect ELISA	ICR mice	200 µg	(272)
	Hepatoprote-ctive	Sd	Decoc.	EtOH	Liver function markers and histopathological study	Wistar-albino rats	125 and 250 mg/kg	(33)
	Antioxidant	Sd	Decoc.	EtOH	Antioxidant enzyme levels	Wistar-albino rats	125 and 250 mg/kg	(33)
	Antiosteopo-rotic	Sd	----------	95 % EtOH	alkaline phosphatases activity Alamar-Blue cell proliferation assay Reporter assays	UMR-106 cells	-------------	(22)
	Improve erectile dysfunction	Sd	----------	----------	Radioimmunoassay	New Zealand white rabbits	1-5 mg/mL	(288)
	Anti-inflammatory	Sd	----------	80 % EtOH	Griess assay ELIZA	Mouse microglia line BV-2 cells	-------------	(255)
	Anti-apoptosis	Sd	----------	95 % EtOH	Annexin V- FITC method	SD rats	-------------	(303)
	Effect on Melanogenesis	Sd	Hot Ex	EtOH water	Melanin contents and tyrosinase activity	B16F10 mouse melanoma cells Zebrafish	-------------	(160)
	Cytotoxic	WP	----------	----------	Methyl tetrazolium bromide test	Human Acute Lymphoblastic Leukemia Cell Line	3 µg/mL in 24 hours	(267)
*C. japonica*	Antihyperte-nsive	Sd	----------	EA MtOH	Plasma ACE activity	Rats	400 mg/ml	(226)
	Melanogenesis inhibition	Sd	Hot Ex	Water	Tyrosinase activity assay melanin contents cAMP assay Western blot analysis	B16F10 mouse melanoma cells (CRL 6323)	-------------	(69)
	Memory enhancing	Sd	Sonicat.	Water	Novel object recognition test The step-through passive avoidance test Immunohistochemistry	ICR mice	50 and 100 mg/kg/day	(315)
	Melasma elimination	AP	Heating	Water	Melasma Area Severity Index degree of hyperpigmentation	Patients	4.8 g/day	(311)
*C. australis*	Hepatoprot-ective	St	Soxhlet	EtOH	hepatic injury markers	Wistar rats	125 and 250 mg/kg	(70)
*C. europaea*	Antibacterial	WP	Shaking	EtOH	Agar well method	*S. aureus * *E. coli*	20 mg/mL	(263)
*C. planif'lora*	Antidepre-ssant	AP	----------	----------	Triple-blind controlled clinical trial	Depression patients	500 mg capsule	(308)
*C. campestres*	Analgesic	WP	----------	95 %EtOH	Writhing Test	Albino mice	50 and 100 mg/kg.	(47)
			Cold Macera.	MeOH	Writhing Test Heat conduction method	Swiss Albino mice	400 mg/kg	(46)
	Antipyretic	WP	----------	95 % EtOH	electric thermocouple	Albino mice	50 and 100 mg/kg	(47)
	Antiiflammatory	WP	----------	95 % EtOH	Volume plethysmographically	Albino mice	100 mg/kg	(47)
	CNS-depressant	WP	----------	95 % EtOH	Behavioural study	Albino mice	50 and 100 mg/kg	(47)
	Anticancer	WP	----------	EA MeOH	---------------	Hepatocellularcarcinoma cell line	-----------	(270)
		AP	Macera.	MeOH	RT PCR analysis	MCF 10A, MCF-7 and MDA-MB-231 cell lines	-----------	(271)
	Antiviral	AP	Shaking	MeOH	RT-PCR analysis	Peripheral blood mononuclear cell	1000 mg/kg	(264)
	Hepatopr-otective	WP	----------	75 % EtOH	Biochemical parameters and histological	Mice	20, 100 and 500 mg/kg	(104)
*C. racemosa*	Antimicro-bial	WP	Percola.	70 % EtOH	Dilution in a liquid medium	*S. aureus*	2 mg/ml	(214)
*C. pedicellata*	Anti-obesity	WP		EtOH	Biochemical measurements	Albino rats	400 mg/kg	(26)
	Antioxidant	Sd	Soxhlet Macera.	MeOH	DPPH assay	------------------	--------------	(232)
	Antibacterial	L	Decoc. infusion	Water	-----------------	*Xanthomonas campestris*	--------------	(74)
		L St Fr	----------	MeOH	Agar well diffusion method	*S. aureus * *P. aeruginosa* *K. pneumonia * *A. baumannii*	100 μL	(168)
	Antifungal	L St Fr	----------	MeOH	Agar tube dilution method	*Aspergillus fumigatus, Aspergillus flavus* *Rhizopus oryzae*	67 μL	(168)
	Cytotoxic potential	L St Fr	----------	MeOH	Brine shrimp assay	--------------------	--------------	(168)
	anti-inflammatory	L St Fr	----------	MeOH	Albumin denaturation, membrane stabilization proteinase inhibitory assays	--------------------	200 μg/mL	(168)
*C. * *epithymum*	Hepatoprotective	WP	Soxhlet	MeOH	Blood serum parameters	Wistar albino rats	400 mg/kg bw	(182)
*C. * *kotschyana*	Anticancer	Sd St	Decoc.	2N HCl EA	MTT assay Annexin V	MCf-7 cell line	100 μg/ml	(77)
*C. mitraeformis*	Antioxidant	St	Hydro. dil	n-hex	DPPH assay	---------------------	--------------	(78)
	Antimicro-bial	St	Hydro. dil	n-hex aceton	broth microdilution method	*C. michiganensis* *E. carotovora* *P. syringae*	--------------	(78)
*C. arvensis*	Analgesic activity	WP	----------	n-hex, DCM EA MeOH water	Writhing test	Swiss albino mice	100 mg/kg	(257)

WP whole plant; AP aerial parts; Fl flower; St stem; Sd seed;Fr fruit; L leaves; Aq. aqueous; MeOH methanol; EtOH ethanol; Pet. eth petroleum ether; n-hex n-hexan; EA ethyl acetate; DMC dichloromethane; CF chloroform; Macera maceration; Decoc decoction; Hydro. Dil hydro distillation; Percola percolation; Sonicat sonication; Hot Ex hot extraction; Suc. Ex successive extraction

**Medicinal uses**

The local inhabitants of rural areas are aware of inherent properties of various plants. They preferentially use these herbs and their products to treat multiple types of diseases due to their handiness and low cost (117). Potentially useful plants have been acknowledged and sequentially conveyed throughout the centuries in all societies. Some of them are used through self-medication, while others are recommended by traditional healers (118). Plant utilization as medicine ranges from the direct administration of the leaves, seeds, barks, roots, and stems to the extracts and decoctions from different parts of the plants (119). 

Many *Cuscuta* species being rich sources of diverse phytochemicals are popular components of various folk medicinal systems. *Cuscuta* species are used in traditional medicine as a purgative, diaphoretic, anthelmintic, diuretic, and tonic as well as a treatment for itching and bilious disorders (120, 121). Seeds, stem, and whole plant are utilized as prescription to treat different types of ailments. Medicinal uses of several parts of *Cuscuta* members are given in Table 2. 

*C. reflexa* is a treasured medicinal herb and widely used in conventional medicinal system of various Asian countries including China, India, Bangladesh, and Thailand for treating multiple disorders (122). It is called a miracle therapeutic plant in the ethnobotany, and a wide array of chemical compounds has been isolated with diverse medicinal properties (123). *C. reflexa* whole plant is used to treat conjunctivitis, respiratory disorders, piles, ulcers, and stomach problems (124). The paste of whole plant mixed with latex *Carica papaya* causes abortions (125). In rural areas of India its juice is used against jaundice. Paste of plant is effective to treat headache, gout, and rheumatism (67, 126-128). Plant juice mixed with other decoctions is purgative. Seeds of *C. reflexa* are carminative, anthelmintic, alterative, emmenagogue, sedative, and diuretic. It is effective against warts (116, 129). Leaves are used to treat eczema, scabies, cold, and to induce sterility (18, 130). Rabha tribes of west Bengal use the whole plant to treat leucorrhoea (131). It is applied internally to cure protracted fevers and externally on itchy skin. The plant is frequently used in Ayurvedic medicine to give relief in urinating difficulties, muscle pain, and coughs (132, 133). Pills prepared from the dried plant are used for treatment of tuberculosis (89). Its stem is a blood purifier, good for brain and fever (134-135). Tribal people use its various parts to treat fits, insanity, melancholy, and to control fertility (15). It is commonly used in veterinary medicines as poultice and sprains. The powder is used as astringent and diaphoretic for cattle (136-137).

*C. reflexa* stems are crushed with *Clerodendrum viscosum* leaves and fed to cattle to treat anthrax (138). The plant is used for skin infections and dandruff (139-140). The paste of whole plant with *Achyranthes aspera* is used to control excessive bleeding during menstruation (141). It is also used for treatment of bone fracture and body pain (142). In folk medicine of Bangladesh, it is used to cure tumors (17). The Tripura community of Bangladesh and Satar tribes in Nepal use this plant to cure edema, body ache and for maintenance of liver function. It is used for treating constipation, spleen diseases, diarrhea, and inflammation. Paste mixed with sesame oil is applied for curing hair fall. The decoction of stem is used to cure diarrhea, cholera, and asthma, while decoction of seeds causes depression, nausea, and vomiting (29, 143-145). Whole plant powder is used to treat jaundice by tribal people of nallamalais in Andhra Pradesh (146).

It is also used as expectorant, aphrodisiac, is useful in vomiting, and purifies the blood (32). *C. reflexa *is an essential constituent of several medical compositions, which are used in the treatment of migraine, headache, chronic catarrh, epilepsy, amnesia, and to prolong fever (27, 147-148). Maceration of whole plant is used to treat infections (149). The whole plant is also useful in cephalagia, paralysis, stomach pain and helminthiasis (89, 150). 

*C. chinesis *Lam. also known as Chinese dodder or* Tu-Si-Zi, *also has a wide range of uses. It has been mentioned in various old Chinese scripts and recommended by many herbal practitioners (68). Besides China it is also a famous prescription in many other countries. In Pakistan dressing made of plant is used on painful inflammations. Moreover, paste is useful for chronic ulcers and wounds (151). In traditional Indian system, leaves and stems are used to enhance lactation (152). In Vietnam people use whole plant in back pain and constipation (153). In Korea, seeds with other herbal prescriptions are effective to improve sexual function and health (154). Stem paste of *C. chinensis* is applied to fractured bone to promote the joining (155). Whole plant juice is used to treat inflammation and jaundice (19, 156). A lotion prepared from stem is used to treat sore heads and inflamed eyes. It has been found useful in the treatment of impotence, nocturnal emissions, dizziness, lumbago, leucorrhoea, decreased eyesight, abortion, and chronic diarrhea (133). *C. chinensis* is used in treatment of mania, epilepsy, and insanity (157). Its stem and seeds are considered tonic, expectorant, purgative, sedative, diuretic, diaphoretic, carminative, anthelmintic, and advantageous in muscles and joints pain (158-159). Prescriptions containing *C. chinensis* are used to treat impairment of sexual function, cure cardiovascular diseases and osteoporosis, treatment of premature ejaculation, to treat lower abdominal and back pain, infertility, wet dreams, impotence, urinary retention, and urinary incontinence (68). It is also used to cure melisma, freckles and considered as anti-dandruff agent (160-161). 

*C. epithymum* is a mild diuretic and used to treat sciatica and scurvy. The fresh plant is applied to the skin against scrofula derma and scleroderma. It is associated with the health of liver and kidneys and used in various formulas. It is considered a mild laxative (162-163). The whole plant is dried and used as astringent and detersive (75). Whole plant decoction of *C. campestris* is used as purgative and poultice (105). The sap of *C. tinctoria* is used to cure ringworm and warts (79). Juice of *C. gigantea* plant is famous as an anti-poisonous agent (140, 164). The sap of *C. europaea* is used as a carminative, and the extract is applied to treat psoriasis (165). Seeds and vegetative parasitic plant is used as laxative, diuretic, and pain reliever and is poisonous. The juice is used for skin treatment (166-167). *C. capitata *whole plant reduces irritation of bladder and improves urinary function (80). *C. hyaline *is used to treat chest pain (20, 24). Its infusion is used as sores washer and to prevent abortion (21, 73). It is antiulcer and used against culex mosquito. *C. australis* is used as laxative, anthelmintic, astringent, for treatment of sores, measles and as kidney and liver tonic, emollient, sedative, and sudorific (23).

Leaves of *C. japonica* are considered antihypertensive (93). The sap of *C. kilimanjari *collected from stems is directly installed to treat ear, nose, and throat diseases in central Kenya. The whole plant is used to treat stomach ache, edema, agalactia, and in veterinary medicines (23, 76). *C. pedicellate *is used for treatment of obesity, stomachache, to cure wounds, hypertension, as purgative, and anti-inflammatory agent (26, 168). The whole plant of *C.* *planiflora *is carminative and laxative, and the stem is anti-diarrheal (23, 130)*. C. racemosa *has anti-inflammatory and diuretic effects, is also used for stomach and hepatic complaints and treatment of fresh wounds (52).

**Phytochemistry**

Exploration of nature’s garden of medication to expose more acceptable solutions with safety is a subject of interest from prehistoric era as more than half of world population still relies on medicinal plants to sustain life. The capability of these odds to appease and treat various diseases and infirmity is undoubted. The curative plants are extensively used in pharmaceuticals, food industry mostly as functional food, agricultural, and cosmetics. Various herbs, their extracts, and prescriptions are loaded with different biologically active constituents particularly alkaloids, steroids, saponins, flavonoids, and terpenoids that are responsible for their therapeutic outcomes (27, 184-189). Phytochemical screening of ever more medicinal plants is extremely momentous in detecting and identifying innovative sources of healing as well as commercially important compounds (190).

Genus *Cuscuta* is rich in many phytoconstituents representing a varied spectrum of secondary metabolites including flavonoids, alkaloids, lignans, polysaccharides, steroids, volatile oils, and resin glycosides (191-199). In a comparative study it was suggested that the plants in the *Cuscuta* species are blessed with almost same soluble phenolic secondary metabolites as Chlorogenic acid,3,5-dicaffeoylquinic acid, 4,5-dicaffeoylquinic acid, hyperoside, quercetin, astragalin, kaempferol-3-O-galactoside, and quercetin-3-O-glucoside but with varying quantities (200).

Chemical constituents of *Cuscuta *species are host-dependent. For instance, a large number of alkaloids identified in these parasitic plants are the same as those found in their alkaloid containing hosts except a very few (201). These species can synthesize flavonoids, while the study of relation between flavonoids of host and parasite is under consideration. Preliminary determination indicates that ﬂavonoid content of various *Cuscuta* samples growing on different hosts is quite different (202). The most thoroughly characterized species of this genus are *C. reflexa *and *C. chinensis *(67-68, 203).

Essential component of many medicinal compositions of *C. reflexa* has an extensively varied array of phytochemicals identified as phenolic compounds, flavonoids, alkaloids, phytosterols, amarbelin, betasterol, stigmasterol, glycosides, saponins, cuscutine, myricetin, dulcitol, coumarin, cuscutamine, luteolin, bergenin, proteins, fixed oils, fats, and carbohydrates (27, 67, 204). 

This genus is a source of many novel metabolites. Qualitative analysis of methanolic extract of *C. reflexa* isolated two new compounds named as 7’-(3’,4’-dihydroxyphenyl)-N-[(4-methoxyphenyl)ethyl]propenamide and 7’-(4’-hydroxy,3’-methoxyphenyl)-N-[(4-butylphenyl)ethyl]propenamide (38). From aerial parts of same plant two novel tetrahydrofuran derivatives, namely Swarnalin and Cis-swarnelin were separated (205) while a flavanon, reflexin chemically named as 5-hydroxy-7-methoxy-6-(2,3-epoxy-3-methylbutyl)-flavanone, was isolated from the stem (206). Moreover, 3’-methoxy-3,4’,5,7-tetrahydroxy flavone and 3’-methoxy-4’,5,7-trihydroxy flavone-3-glucoside were isolated from whole plant (207). An antiviral protein with molecular weight about 14,000–18,000 Daltons was separated and evaluated against several isometric and anisometric viruses (208).

Phytochemical investigations of *C. chinensis* have shown that flavonoids, alkaloids, poly-saccharides, steroids, lignans, and volatile oils are mostly reported in its various parts (68). The active moieties responsible for various pharmacological activities of the *C. chinensis* mostly include flavonoids, lignans, quinic acid, and polysaccharide. Flavonoids are the prime biologically active components in *C. chinesis*. Additionally, quercetin, kaempferol, and hyperoside can serve as an index to evaluate the quality of the crude drug (209). 

*C. chinensis* extract afforded four new lignans cuscutoside A (2′-hydroxyl asarinin 2′-O-β-D-apiofuranosyl-(1→2)-β-D-glucopyranoside), cuscutoside B (2′-hydroxyl asarinin 2′-O-β-xylopyranosyl-(1→6)-β-glucopyranoside), cuscutoside C (2′-hydroxyl asarinin 2′-O-β-D-glucopyranoside), cuscutoside D (2′-hydroxyl asarinin2′-O-β-D-apiofuranosyl-(1→2)-[β-D-glucopyranosyl-(1 → 6)]-β-D-glucopyranoside) and neo-sesamin (188, 193, 210). *C. chinensis* and *C. australis* are used to prepare the famous Chinese herbal prescription Tu-Si-Zi. Phytochemical analysis was done to compare the phenolic constituents of both plants. Principal compounds of *C. australis *were kaempferol and astragalin while hyperoside was predominant in *C. chinensis* (211). Several Phytoestrogens were isolated and identified from *C. chinensis. *Ethanolic extract of seeds afforded three new lignans named cuscutaresinols A−C (212). In another investigative study, four new glycosidic acids called cuscutic acids A-D were isolated from the alkaline hydrolysate of the ether-insoluble resin glycoside (191)*. *Up till now bulk of the phytochemical investigations on *C. chinensis *targeted the seeds while other parts of the plant have had much less attention by the researchers. 

An ether insoluble resin glycoside fraction was separated from seeds of *C. australis* and identification and characterization of resin matrix revealed the presence of three new glycosidic acids, cuscutic acids A_1_−A_3_ (213). *C. racemosa* like other species of the genus offers flavonoids as the chief constituent along with tannins. In another experiment alkaloids, flavonoids, tannins, and saponins have been identified (52, 214). 

**Pharmacological attributes**

Impressive medicinal background of *Cuscuta *species has attracted the attention of many pharmacological researchers. A good deal of biological attributes has been studied and is listed in tabular form in Table 4.

**Antioxidant**

Medicinally important plants are endless reservoirs of antioxidants that enhance the antioxidant capacity of the body, which lead to a reduced risk of many diseases (234-235). Although a diverse population of synthetic analogs is commercially available due to side effects (liver impairment and carcinogenesis) blind reliance on these formulations has been over. Therefore, plants can play a key role to fulfill prerequisite for exploration of effective, biocompatible, and economic antioxidants (236).

Many investigators have employed different qualitative and quantitative approaches to detect antioxidants in various *Cuscuta* species. Stem collected from different hosts and extracted with various solvents (100% methanol, 80% methanol, 100% ethanol, 80% ethanol, water, and n-hexane) were analyzed for quantity of phenolics and flavonoids content. Their antioxidant capacity was measured by using a variety of assays including reducing power, DPPH scavenging activity, percent inhibition of linoleic acid peroxidation and δ-tocopherol. It was observed that there was a strong correlation between amount of total phenolics and antioxidant capacity (13).

*C. reflexa *has been reported for its antioxidant potential (37, 237). Free radical scavenging capacity of methanolic extract of *C. reflexa *was evaluated by DPPH and reducing power assays. Results of DPPH assay, illustrated as IC50 value demonstrated its antioxidant activity 359.48 μg/ml as compared to 9.22 μg/ml value for ascorbic acid used as standard. The reducing power of extract was found dose-dependent and increased by increasing concentration (35). Ethyl acetate fraction of ethanolic extract of *C. reflexa* was significantly antioxidant. Activity may be related to presence of flavonoids, alpha tocopherol, and rutin, which were confirmed in preliminary phytochemical screening (238).

Seed oil of *C. pedicellata *was extracted with petroleum ether (pet. ether) and lipid contents were saponified to separate unsaponifiable materials and fatty acids. The extract was fractionated by using various solvents, and antioxidant activity of all extracts (pet. ether, unsaponified, fatty acids, 70 % methanol, ethyl acetate, and chloroform) was appraised by DPPH free radical assay. The methanol extract was found most potent (230).

In another study, a correlation was established between antioxidant activity and total phenolic content of aerial parts of three Iranian *Cuscuta *species. *C. approximate, C. monogyna *and *C. campestris* were estimated by using DPPH microplate method. The highest concentration of phenolic compounds was found in *C. monogyna *and *C. approximata*. TPC of plant methanolic extracts was determined. Methanolic extracts of *C. approximata *and *C. monogyna *contain highest amounts of total phenolic, 56.67 mg/g and 49.59 mg/g, respectively, while antioxidant potential was in the order *C. monogyna ****˃ ****C. approximate* ˃ *C. campestris *(14).

Ethyl acetate fraction of ethanolic extract of *C. chinensis* seeds possesses strongest antioxidant effect with kaempferol and quercetin as its main constituents. It hunts free radicals and inhibits liquid peroxidation (198, 239). The same fraction of methanolic extract was ascertained as an effective antioxidant by DPPH free radical scavenging assay (222). Moreover, aqueous extract of *C. chinensis* can protect murine osteoblastic MC3T3-E1 cells against tertiary butyl hydroperoxide induced injury because of its oxidation stress management potential and functioning against mitochondria-dependent pathways (240). In another experiment, flavonoids of *C. chinensis* were evaluated for their protective effect against oxidative stress. The survival rate of PC12 cells having H_2_O_2_-induced apoptosis was measured. The protective effect was possibly due to scavenging of reactive oxidative species and enhanced activity of antioxidant enzyme (241). Essential oils and carotenoids separated from *C. mitraeformis *also showed antioxidant activity (78). These results suggest that *Cuscuta *plants are enriched with highly important natural antioxidants that may be used in development of functional foods and drugs effective against diseases caused by oxidative stress. Isolation, identification and possible synergism among various components may be the subject of interest for further studies.

**Hepatoprotective**

Anti-hepatotoxic drug designing is a major thrust area seeking the attention of natural product researchers because synthetic formulations have serious side effects. *C. epithymum *is traditionally used as a liver tonic. *C. epithymum *whole plant extracted in methanol exhibited appreciably high hepatoprotective effect against CCl_4_ induced hepatotoxicity in albino rats. Elevated serum aspartate aminotransferase (AST), alanine aminotransferase (ALT), alkaline phosphatase (ALP) and total bilirubin have confirmed hepatic damage after CCl_4 _administration. *C. epithymum *prevented the toxic effect in both anticipatory and curative models, which may be due to the presence of various bioactive moieties, including phenolics, flavonoids, and alkaloids (185). 

Many investigators have studied the curative effect of *C. reflexa *against liver damage induced by cisplatin, paracetamol, carbon tetrachloride, ethanol, isoniazid, and rifampicin. Various biochemical measurements were observed including ALT, AST, ALP, and total bilirubin before and after the administration of *C. reflexa *extract. It improved liver function by significantly reducing the serum ALT, AST, and ALP levels in affected rats comparable to standard. Histopathological examination of liver section supports the results (32, 242, 243).

Ethanolic extract of *C. australis *also appeared as liver protector against acetaminophen intoxication in an animal model. Two groups of rats were intoxicated on day eight after receiving doses of *C. australis *seed and stem extract separately for seven days. In untreated rats, severe periportal hepatic necrosis, considerably raised serum liver damage markers, noticeably augmented lipid peroxidation and suppressed liver antioxidant enzymes activities were witnessed. Comparative evaluation of seed and stem extract proves that stem is a more potent hepatoprotective counterpart than seed (70).

Seeds of *C. chinensis* are commonly employed to nourish and improve hepatic disorders in China and various other Asian countries. Oxidative stress can stimulate the development of acetaminophen-induced hepatotoxicity. Liver protecting and antioxidant activities of ethanolic and aqueous extracts of *C. chinensis* on acetaminophen-induced hepatotoxicity in rats. Ethanolic extract showed a significant hepatoprotective effect at an oral dose of 125 and 250 mg/kg confirmed by the measurement of various parameters and observation of liver histopathology. Comparatively same doses of the aqueous extract were found ineffective rather; it resulted in further hepatic deterioration (33). *C. chinensis* nanoparticles were found more effective in this regard (198, 239). Thus, from the above findings it can be observed that many *Cuscuta* species are promising hepatoprotective agents supporting the claims of traditional healers. Further investigations on chemical components are needed to pinpoint the findings.

**Antidiabetic**

Diabetes mellitus is becoming a growing threat for a vast population in almost all countries of the world due to a sluggish lifestyle leading to reduced physical activity and increase in obesity (244). Methanolic and chloroform extracts of *C. reflexa *whole plant exhibited significant hypoglycemic activity at doses of 50, 100, and 200 mg/kg body weight. Oral glucose tolerance test was used to estimate the effect in glucose-loaded Long Evans rats (50). Administration of methanolic extract *C. reflexa* to glucose-loaded mice led to notable reductions in blood glucose and improved metabolic alterations, thereby justifying its traditional folkloric claims (89, 245).

Antidiabetic activity of *C. chinensis* was evaluated in dexamethasone-induced insulin-resistant human liver carcinoma (HepG2) cells (246). *C. chinensis* polysaccharides can reduce blood sugar level in type-2 diabetes. Efﬁcacy was tested on alloxan-induced diabetes in a mice model. Orally administrated doses of 300 and 600 mg/kg remarkably decreased the elevated fasting blood glucose (247-248). In a similar study, oral administration of 200 and 400 mg/kg polysaccharides significantly lessened blood glucose along with glycosylate serum protein (249). A Chinese herbal prescription, Zhujing pill, having more than 50 % *C. chinensis* protected retina of diabetic rats, possibly through its antioxidation and anti-inflammatory effects (250). Recently mechanism of hypoglycemic activity of *C. chinesis *on type 1 diabetic disease was investigated using a rat model. Daily administration of *C. chinesis *extract returned fasting serum insulin and fasting blood glucose to normal value by upregulating the gene expression of hepatic and pancreas genes (251). It is crucial to continue the exploration of hypoglycaemic effect of more plants as these are blessed with similar chemical profile.

**Anti-inflammatory**

Inflammatory reactions play a decisive role in different phases of pathogenesis of cancer. So, there may be an assumption that anti-inflammatory drugs can induce apoptosis in cancerous cells and may be equally beneficial as preventive measure and therapy (252). Aqueous and alcoholic extracts of stem of *C. reflexa* and its ethyl acetate fraction showed remarkable anti-inflammatory activity in *in vitro* and *in vivo* tests. Inflammation was induced by various chemicals like histamine and lipopolysaccharide. It was observed that extracts inhibited inflammatory responses that can be related to the presence of flavonoids, phenols, and polyphenols in this plant (43-44, 253). *C. reflexa* significantly suppressed inflammation by reducing edema volume up to 80 % in rats as compared to standard 96.36 % (254).

*C. campestris* markedly inhibited carrageenan-induced edema in rats by oral pretreatment with 100 mg/kg extract (47). *C. chinensis,* by suppressing the inflammatory responses showed the potential for treatment of brain inflammation (255). Moreover, λ-carrageenan-induced paw edema treatment by using the methanolic extract of *C. chinensis* seed in mice, also confirmed its anti-inﬂammatory effect ( ). *C. pedicelleta *and *C. arvensis *were found effective against inflammation (168, 257). Further studies must be conducted to clarify the mechanism and to figure out the active principle behind the activity.

**Antibacterial, antifungal, and antiviral**

Continuous and urgent exploration is required for new antimicrobial agents with new compositions and diverse mechanisms of action to overcome antimicrobial modifications (9). Methanolic extract of *C. reflexa* was found significantly active against a broad spectrum of bacterial species including *S. aureus, P. aeruginosa, S. dysenteriae, S. boydii, *and* E. coli* with impressive zone of inhibition (27, 258-260).

*Xanthomonas campestris (XC) is *a widely spread infectious agent causing a huge loss in food crops with visible symptoms and leave shedding. Aqueous decoction and infusion extract of *C. pedicellata *were evaluated for antibacterial activity against diverse pathovars of *XC *using *in vitro* well diffusion method. Inhibition zone diameter was observed from 1.0 to 5.0 cm (74). The methanolic extract also showed promising high antimicrobial activity (168). *C. australis* is another species having notable antibacterial effect. The 50 % methanolic extract was fractionated by hexane, ethyl acetate, and butanol with various polarities. All fractions were tested against fungal, yeast and various Gram-positive and Gram-negative bacteria. All extracts except n-hexane were found effective against different species (261). Additionally, methanolic extract of *C. epithymum *was also significantly active against* Bordetella bronchiseptica *demonstrating zone of inhibition from 10–14 mm (262). *C. europaea *was active against *Staphylococcus aureus* even higher than standard drug Amoxicillin. These results lead toward the concept that this plant can be used as a safer option against this microbe (263). Recently essential oils and carotenoids separated from *C. mitraeformis *were found antibacterial (78). 

In addition to many other species of genus *Cuscuta,* *C. racemose* offers flavonoids as chief metabolites. Slightly positive antimicrobial activity of this plant was observed against *S. aureus* using dilution in a liquid medium method. Minimum inhibiting concentration was 2.0 mg/ml. Phenolic compounds are documented as antimicrobial substances. So, the activity can be ascribed to the flavonoids and tannins in the plant (52).

Several secondary metabolites like flavans, flavones, and quinic acid derivatives have been found active against HIV infection. Crude aqueous extracts of *C. reflexa* exhibited anti-HIV activity. Virus inhibition may be attributed to the combinatory effects of nine closely related compounds (49). An antiviral protein with significantly high inhibiting property was isolated from the aqueous extract of *C. reflexa* (219). Methanolic extract of *C. campestris *showed weak anti-HIV activity (264). A number of species have been found effective against microbes. It is recommended that further studies with isolated components instead of extracts may be more useful to identify the active compounds.

**Antitumor effect**

Some species of the genus *Cuscuta *afford alkaloids with indolic nuclei that are considered potential antitumor substances. *C. chinensis* is a popular antitumor prescription in the Unani medicine system. Oral administration of the plant extract at a dose of 1 g/kg noticeably delayed the appearance and growth of skin papilloma and reduced the chances of carcinoma (30). Anticancer activity of *C. chinensis* has been evaluated by several pharmacological studies using a variety of cell lines. Results prove that it can act as an integrative approach to encounter ever-growing disease management (22, 31, 265- 267).

*In vivo* anticancer potential of *C. reflexa* was determined by using murine models. Alcoholic extract and its chloroform fraction were found more potent. It showed highest toxicity against human breast cancer cell lines. Similarly, chloroform part of extract of alcohol showed considerable tumor growth inhibition, which reveals that these extracts interfere in cell proliferation to inhibit cancer (15). It can induce apoptosis in Hep3B cells (253). Phenolic components isolated from *C. reflexa *were also assessed in HCT116 colorectal cells amongst which 1-O-p-hydroxycinnamoylglucose could show considerable anticancer activity (10).

The seed extract of *C. kotschyana* induced apoptosis in breast cancer cell line (MCF7) (77). As the major active phytoconstituents of *C. kotschyana* are flavonols, quercetin, and kaempferol (231) and quercetin has been found to reduce cell viability of quite a lot of cancer cell lines *in vitro* (268-269). Therefore, these facts are consistent with results that the exposure of MCF7 cells to *C. kotschyana* considerably reduced viability (77). 

*C. campestris *also has anticancer agents (270). Detection and evaluation of phytochemicals suggested that eugenol epoxide, lutein epoxide, and lupeol epoxide formed the most active fractions and exhibited the cytotoxic effects against breast cancer cells (271). In a recent effort, efficacy of a Korean herbal formula Ga Gam Nai Go Hyan containing *C. japonica* against benign prostatic hyperplasia was evaluated. This herbal prescription significantly decreases prostate weight by regulating inflammatory responses and apoptosis (92). There is need to develop new technologies such as nanoparticles to improve the therapeutic effect of compounds isolated from these plants. Further efforts may be used to design sustained and targeted drug release systems to improve avoiding side effects. 

**Immunological effects**

Ethanolic extract of *C. chinensis* showed considerable adjuvant potentials towards cellular and humoral immune responses in mice models and can be used as vaccine adjuvants. Extract enhanced specific antibodies (IgG, IgG1, and IgG2b) to a noticeably high level by affecting Th1 and Th2 cell functions (272). Dendritic cells play a key role in regulating immune responses and are a major target to develop immune modulators. n-butanol and methanol extracts exhibited the immunosuppressive effect on dendritic cells. Kaempferol was identified as the main flavonoid of methanol fraction. Results suggest that kaempferol has potential to treat chronic inflammatory and autoimmune diseases (273). Furthermore, aqueous extract of *C. chinensis *also improved the immune responses (274). *C. chinensis* can protect against tertiary butyl hydroperoxide induced murine osteoblastic MC3T3-E1cell injury. Aqueous extract of seeds protected cells in a dose-dependent manner by modulating the oxidative stress-induced apoptosis probably owing to its antioxidant potential (240). *C. australis* may act as an immunopotentiator for mammals by increasing the percentage of phagocytosis (275). *C. australis* hyperoside can decrease T or B lymphocyte proliferation and phagocytic activity of the peritoneal M and mediate immune regulation (276). 

**Effect on the neuronal system**

*C. chinensis* can act as a neuroactive agent and improves memory by inducing cell differentiation. Glycoside of the plant induced neuronal differentiation in rat pheochromocytoma PC12 cells (277). In another experiment, *C. chinensis* improved memory and inhibited acetylcholinesterase activity in scopolamine-induced dysmnesia mice (278). Oral administration of its aqueous extract recovered the ischemia-induced lethal damage of neurons and prevented learning disability (51). A traditional Chinese formula Wu-Zi-Yan-Zong containing *C. chinensis *suppresses neuroinflammatory responses and can act as an effective therapeutic agent to prevent and treat neuroinflammatory defects (279).

**Anti-aging activities**

*C. chinensis* is an important antiaging prescription of the Chinese herbal medicinal system. Various experimental efforts have been employed to test the certainty of the claim. Polysaccharides of *C. chinensis* can exhibit anti-aging effects by scavenging free radicals and opposing lipid peroxidation (280). Ethanolic extract of *C. chinensis* signiﬁcantly suppressed the non-enzymatic glycosylation of D-galactose-induced rat aging model (281). Various research reports obviously show that it can regulate immune responses, prolong cell cycle, positively affect body metabolism, improve physiology of internal body organs, and stress management, which proves its anti-aging effects (282). 

**Antihypertensive**

Ethanolic extract of *C. reflexa *decreased arterial blood pressure and heartbeat rate in Pentothal anesthetized rats. Experimental data indicated that it is a non-specific depressant on all the isolated tissues tested (283). In the course of experiments, ethyl acetate fraction of *C. japonica* exhibited distinctive angiotensin-converting enzyme (ACE) inhibition at a dose of 400 mg/ml. Four caffeoylquinic acid derivatives were isolated from the active fraction having inhibitory effects on ACE activity. Presence of these metabolites, at least in part is responsible for the antihypertensive activity extract (229). 

**Anti-osteoporotic activity**

*C. chinensis* effectively boasted tissue regeneration of damaged bones by promoting the formation of osteoblasts from their precursor cells (284). It has been demonstrated in an experimental report that aqueous extract of *C. chinensis* signiﬁcantly stimulated the differentiation and proliferation of osteoblasts in rat bone cells, but the osteoclasts activities were inhibited (285-286). Antagonistically antiosteoporotic effect of *C. chinensis* was also observed. Five ﬂavonoids were isolated from which kaempferol and hyperoside were found osteogenic in nature (22). 

**Renoprotective effects**

Aqueous and alcoholic extract of *C. reflexa* exhibited substantial diuretic activity in Wister rats. Total urine volume and Na ^+^, K ^+^ and Cl^−^ concentration was estimated after a dose of 300 mg/kg extract. There was a marked rise in Na ^+^ and K^ +^ excretion (45). *C. chinensis* has been used as a kidney tonic since ancient times. Effect of seed extract on renal function parameters in the rat model having ischemia/reperfusion-induced acute renal failure was studied. Results indicate that *C. chinensis* extract ameliorates renal functions and regulates urine concentration (287).

**Effect on the reproductive system**

*C. reflexa* has an antifertility effect. Methanolic extract arrested the normal estrus cycle and decreased ovarian and uterus weight in adult female mice. Flavonoids are reported as antifertility agents, and *C. reflexa* is rich in flavonoids, so results can be attributed to the presence of such compounds (42). 

*C. chinensis* extract, and its isolations can improve reproductive systems of both males and females. Ethanolic extract of *C. chinensis* induces a relaxing effect on cavernous penile tissue and may improve erectile dysfunction conditions (288). Many formulations of *C. chinensis* with other herbal prescriptions enhanced penile erection, improved erectile dysfunction, infantile uteruses, and motility of sperm (154, 289-291). An herbal formula, KH-204 containing *C. chinensis,* ameliorates erectile dysfunction by its antioxidant and lipid profile improving property (292). Effect of various flavonoids from *C. chinensis* on sex hormones, and prevention of induced and threatened abortion were evaluated by measuring different parameters in a mice model (293-297).

**Anti-mutagenic activity**

Mutations elicit an innate metabolic defect in regular cellular systems and lead to morbidity and mortality in mutated organisms. Therefore, exploration for novel bioactive phytocompounds to encounter promutagenic and carcinogenic effects is a subject of keen interest (298). Preliminary evaluation of methanolic extract of *C. chinensis* suppressed 90 % of mutagenic effect against Trp-P-1 in the Ames test, suggesting it as a potential antimutagenic agent (299).

Mutagenic and antimutagenic effects of *C. reflexa* were also studied by the Ames test against well-known positive mutagens including 2-aminofluorine, 4-nitro-o-phenylenediamine, and sodium azide in Salmonella typhimurium (TA 98 and TA 100) bacterial strains. The extract revealed noteworthy antimutagenic activity against 4-nitro-o-phenylenediamine and sodium azide for *S. typhimurium* strains (122).

**Cardiovascular activities**

The aging process is accompanied by so many diseases like diabetes, cancer, dementia, and cardiovascular diseases. Heart diseases, leading causes of mortality are due to cardiomyocyte apoptosis which play a key role in myocardial damage and heart failure (300-302). In an experiment, effect of polysaccharide of *C. chinensis* was investigated on D-galactose induced apoptosis of cardiomyocytes in an aging rate model. Apoptosis parameter evaluation indicated that polysaccharide extract decreased the apoptosis of cardiomyocytes (303). *C. chinensis* extract can increase coronary blood flow and decrease myocardial oxygen consumption (304).

**CNS depressant activities and anti-depressant activities**

Central nervous system (CNS) disorders comprise 12 % of deaths worldwide and are still a hugely challenging endeavor for health care systems. Plenty of *Convolvulaceae* species, including *Cuscuta *members, are used to treat CNS related diseases traditionally and might be used as alternatives (184).

*C. campestris* affects the CNS action and decreases motor activity of mice sited on a rotarod. Various tests applied indicated the CNS-depressant activity of the extract, which probably seems due to an anesthetizing effect (8, 47). In another experimental trial, methanolic extract of *C. reflexa* served as a good anxiolytic agent in mice at a dose of 400 mg/kg (305). 

*C. chinensis* methanolic extract considerably reduced immobility times estimated by FST forced swimming test, which reveals its antidepressant activity (306). While its aqueous extract shows CNS-depressant activity in mice by reducing motor activity and the tonic/clonic phases of electrically-induced seizures in rats (157). Recently a Chinese herbal medicine, Tiansi liquid, containing *C. chinensis* was evaluated for its antidepressant activity, and possible mechanism of action was predicted by *in silico* study (307). Capsules of *C. planiflora* (500 mg) prepared by a pharmacist were found effective for major depression patients. In a study period of eight weeks depression was measured before and after by Beck Depression Inventory and Hamilton Depression Inventory (308).

**Effect on melanin production**

*C. chinensis* can promote melanogenesis of amelanotic melanocytes and improved the tyrosinase activities (247-248). Furthermore, it significantly enhanced skin melanin and tyrosinase production. It also positively affected vitiligo treatment in guinea pigs (309). Moreover, there is another report on melanogenesis effect of *C. chinensis* seeds aqueous and ethanolic extracts both *in vitro* and *in vivo*. The aqueous extract showed inhibitory effect on tyrosinase, while the ethanolic extract displayed the opposite effect in tyrosinase activity (160). In a similar study aqueous and ethanolic extracts of *C. chinensis* seeds significantly influenced the melanogenesis by regulating the activity of tyrosinase (310). Consumption of the *C. chinensis* extract with milk reduced the melatonin synthesis and thus ameliorated the elimination of melasma (311).

*C. japonica* has an inhibitory effect on mushroom tyrosinase activity (312). It can also be used to improve hyperpigmentation. It was ascertained by the treatment of alpha-melanocyte-stimulating hormone-induced melanogenesis with aqueous extract in mouse melanoma cells (69).

**Anti hair fall and anthelmintic activities**

Hair loss is a feared side effect of chemotherapy and creates a psychologically distressing condition among millions of men and women due to the deprivation of their major esthetic display feature. Plants as hair growth promotors have found their use in almost all traditional medicinal systems. *C. reflexa* extract is useful in the treatment of alopecia by promoting hair growth (40, 313). Methanolic extract of *C. chinensis* was used as an anthelmintic drug against *Dactylogyrus intermedius* in goldﬁsh (314). 

**Analgesic and psychopharmacological**

*C. campestris* has analgesic properties. The whole plant grown on *Nerium indicum* was studied. Acetic acid induced writhing test and heat conduction method were used to study the described activity in an animal model. A dose of 400 mg/kg methanolic extract gave significant results as compared to standard Diclofenac sodium (46). In a similar experiment, protecting response against p-benzoquinone-induced writhing was studied by giving a dose of 100 mg/kg to mice, which suggested the analgesic activity of the extract (47). *C. chinensis *also has a pain-relieving ability which was examined by using acetic acid-induced writhing response and formalin-induced paw licking method (256). 

Petroleum ether extract of *C. reflexa* noticeably decreased the spontaneous activity and behavior profile of Swiss albino mice. Steroids, the major constituents of the extract may be responsible for such changes (39). *C. Japonica* treatment improved the cognitive function of mice in a dose-dependent manner. Novel object recognition and passive avoidance test proved that it might improve learning and memory (315). 

**Antipyretic and antiulcer**

Antipyretics agents lessened the body temperature in fever. Efficacy of *C. reflexa* as an antipyretic agent was confirmed in yeast induced pyrexia in rats. Aqueous and ethanolic extracts were both found active and started rectal temperature decline after three hours of dose. A dose of 400 mg/kg weight reduced the elevated temperature approximately 83.8 % (ethanolic) and 79 % (aqueous) as compared to the standard drug (96.5 %, Paracetamol) after six hours of treatment (48). *C. campestris* markedly lowered the body temperature of hyperthermic and normothermic mice (47).

Lyophilized raw extract of *C. racemosa* possesses antiulcer activity, which was ascertained by a test showing 44.22 % rate of activity, and 37.05 % rate of cure against acute and sub-chronic models of ulcers, respectively (52).

**Anticonvulsant and anti-obesity**

*C. epithymum* have effective anticonvulsant constituents and delayed the onset of seizure (316). Methanolic extract of *C. reflexa* stem demonstrated preventive effects against convulsion created by chemical agents in mice. Catecholamines levels augmented considerably. After a six-week treatment, γ-aminobutyric acid (GABA) involved in seizure activity was noticeably increased in the brains of mice (317). Ethanolic extract of *C. reflexa* significantly reduced convulsions by delaying onset and duration of seizures in an albino mice model. A dose of 400 mg/kg showed maximum delay in pentylenetetrazole induced convulsions (238).

*C. pedicellata* is widely used for management of obesity. Ethanolic extract of *C. pedicellata* has significantly reduced the bodyweight along with serum lipid profile in high-fat diet-fed rats (26). Recently, polyphenols are reported to possess anti-obesity activity (318).

**Cytotoxicity, insecticidal, antiarthritic, and wound healing activity**

The ethanolic extract *C. reflexa*, parasitizing *Nerium oleander*, exhibited promising cytotoxic activity (208). Lectin-like glycoproteins isolated from *C. europaea* demonstrated the cytotoxic effects of LLP and LLP on C127 and B-16 cells (319). Various extracts of the plant have larvicidal potential against mosquitoes (320). *C. reflexa *protects against arthritis and nephrotoxicity. A dose at 600 mg/kg considerably reduced paw edema and joint swelling up to 71.22 % (321). Aqueous and ethanolic extracts of *C. reflexa *stem at 200 mg/kg and 400 mg/kg were able to heal wounds in a rat model (322).

## Conclusion

*Cuscuta* genus is a rich and diverse source of many valuable chemical components. It is loaded with flavonoids, alkaloids, lignans, polysaccharides, steroids, volatile oils, and resin glycosides. Medicinal importance of its various species is part of prehistoric texts. Traditionally it is considered a miracle genus equipped with broad spectrum of remedial values. Decoctions, extracts, paste, powder, juice, and infusions of different parts of the plants are important herbal prescriptions in traditional medicinal systems.

A lot of experimentation has been employed to verify its phytotherapy as claimed by traditional healers and local inhabitants. *C. reflexa*, *C. chinensis*,* C. pedicellata*,* C. approximate*,* C. monogyna*, *C. campestris, *and* C. mitraeformis* have shown impressive antioxidant activity. *C. chinensis*,* C. australis*,* C. reflexa, *and* C. epithymum *are significantly hepatoprotective in nature*. *Some species of *Cuscuta* including *C. reflexa*, *C. chinensis*,* C. campestris*,* C. japonica, *and* C. *kotschyana have been reported potentially antitumor against various cancer cell lines. Moreover crude extracts and compounds from the various parts possessed antibacterial, antiosteoporotic, anti-inflammatory, antihypertensive, analgesic, anti hair fall, analgesic, and antiestrogenic properties. 

Rich and unrivaled medicinal history demands verification with modern scientific methodologies. Only a few of the species are thoroughly investigated up till now, especially *C. reflexa* and *C. chinensis* out of nearly 170, while the rest of the members are partially or fully undiscovered in terms of phytochemistry and pharmacology. Most of the efforts are limited to *in vitro* and *in vivo* animal models or cell line level. Very few clinical studies are reported in humans. Although a good deal of secondary metabolites with multitudinous pharmacological attributes have been isolated, identified, and characterized but most of the pharmacological investigations are extract-based. Further studies must be conducted to clarify the mechanism and to figure out the active principle behind the activity to use these compounds as leads and template in development of new drugs. 
